# Longitudinal analysis of the peripheral B cell repertoire reveals unique effects of immunization with a new influenza virus strain

**DOI:** 10.1186/s13073-015-0239-y

**Published:** 2015-11-25

**Authors:** Bernardo Cortina-Ceballos, Elizabeth Ernestina Godoy-Lozano, Juan Téllez-Sosa, Marbella Ovilla-Muñoz, Hugo Sámano-Sánchez, Andrés Aguilar-Salgado, Rosa Elena Gómez-Barreto, Humberto Valdovinos-Torres, Irma López-Martínez, Rodrigo Aparicio-Antonio, Mario H. Rodríguez, Jesús Martínez-Barnetche

**Affiliations:** Centro de Investigación Sobre Enfermedades Infecciosas-Instituto Nacional de Salud Pública, Av. Universidad 655. Col. Santa María Ahuacatitlán C.P., 62100 Cuernavaca, Morelos México; Departamento de Virología, Instituto de Diagnóstico y Referencia Epidemiológicos, Francisco de P. Miranda 177. Col. Unidad Lomas de Plateros. Álvaro Obregón, Distrito Federal CP, 01480 México, D. F., México

## Abstract

**Background:**

Despite the potential to produce antibodies that can neutralize different virus (heterotypic neutralization), there is no knowledge of why vaccination against influenza induces protection predominantly against the utilized viral strains (homotypic response). Identification of structural patterns of the B cell repertoire associated to heterotypic neutralization may contribute to identify relevant epitopes for a universal vaccine against influenza.

**Methods:**

Blood samples were collected from volunteers immunized with 2008/2009 trivalent inactivated vaccine (TIV), pandemic H1N1 (pdmH1N1) monovalent inactivated vaccine (MIV) and the 2014/2015 TIV. Neutralization was assessed by hemagglutination and microneutralization test. IgG V_H_ amplicons derived from peripheral blood RNA from pre-immune and 7 days post vaccination were subjected to 454-Roche sequencing. Full reconstruction of the sampled repertoires was done with ImmunediveRsity.

**Results:**

The TIV induced a predominantly homotypic neutralizing serologic response, while the 09 MIV induced a heterotypic neutralizing seroconversion in 17 % of the individuals. Both the 08/09 and the 14/15 TIV were associated with a reduction in clonotypic diversity, whereas 09 MIV was the opposite. Moreover, TIV and MIV induced distinctive patterns of *IGHV* segment use that are consistent with B cell selection by conserved antigenic determinants shared by the pre-pandemic and the pandemic strains. However, low somatic hypermutation rates in IgG after 09 MIV immunization, but not after 08/09 and 14/15 TIV immunization were observed. Furthermore, no evidence of the original antigenic sin was found in the same individuals after vaccination with the three vaccines.

**Conclusions:**

Immunization with a new influenza virus strain (2009 pdmH1N1) induced unique effects in the peripheral B cell repertoire clonal structure, a stereotyped response involving distinctive *IGHV* segment use and low somatic hypermutation levels. These parameters were contrastingly different to those observed in response to pre-pandemic and post-pandemic vaccination, and may be the result of clonal selection of common antigenic determinants, as well as germinal center-independent responses that wane as the pandemic strain becomes seasonal. Our findings may contribute in the understanding of the structural and cellular basis required to develop a universal influenza vaccine.

**Electronic supplementary material:**

The online version of this article (doi:10.1186/s13073-015-0239-y) contains supplementary material, which is available to authorized users.

## Background

Influenza viruses cause seasonal outbreaks and eventually pandemics with a high cost in morbidity and mortality at a global level [1, 2]. Yearly influenza outbreaks are ascribed to the significant mutation ability of the virus. Structural variability of the viral hemagglutinin (HA) (antigen drift) [3], the main viral antigen responsible for interaction with the sialic acid on the host’s cells surface, allows viral escape from neutralization by antibodies induced by previous exposures to a particular viral strain. In contrast, pandemics are caused by the introduction of new viruses that result from genes re-assortment (antigen shift), for which there is no pre-existing immunity (mainly against the new HA); leading to rapid global spread [3]. Despite the enormous variability of influenza viruses, the induction of specific neutralizing antibodies through vaccination continues to be an effective intervention for seasonal influenza prevention, with the constant challenge of renewing the vaccine strain formulation every year in order to counteract the antigen drift, and the limitation of being ineffective in pandemic prevention [4, 5].

Eighteen HA subtypes, with a protein sequence identity between 40 % and 60 %, divided into two phylogenetic groups, have been described [6]. On the virion surface, HA is trimeric, and each monomer contains a globular domain with a high mutation frequency and a stem with a more conserved structure [3]. Both natural infection and vaccination induce the production of neutralizing antibodies mainly directed against the globular domain, known as homotypic neutralizing antibodies, which are incapable of neutralizing other virus subtypes or certain drift variants of the original subtype. However, the presence of antibodies with heterotypic neutralizing capacity – that is, antibodies with the ability to neutralize several strains and subtypes of the virus – has been described in a murine model [7], and more recently in humans [8–10]. Most of these antibodies are directed towards the HA stem, whose sequence is more conserved among virus subtypes and is essential for endosomal virion-host cell membrane fusion [3]. As for why heterotypic neutralizing antibodies do not prevail over homotypic neutralizing antibodies, and why they are not produced in all individuals in relevant amounts to provide protection remain open questions. The answer to these questions would open up the possibility of developing a universal vaccine that may prevent a significant number of virus subtypes, including new variants with pandemic potential [11–14].

Lymphocytes represent a highly diverse population at a cellular and molecular level, which is dynamically modified by selective processes resulting from the individuals’ interaction with their environment [15]. The ability to produce highly specific antibodies against virtually any antigen (adaptive capacity) depends upon the generation of a large diversity of antigen receptors exposed on the surface of B cells (B cell receptor or BCR). Each lymphocyte expresses a single antigen receptor [16]. The diversity of BCRs is generated through the somatic recombination process or V(D)J recombination, which occurs independently of the antigen. Considering the multiplicity of V, D, and J segments encoded in the germ-line, their ability to combine and the junction repair processes (junction diversity), the potential diversity of the B-lymphocyte repertoire has been estimated to be 1 × 10^11^ [17].

The great diversity of the B-lymphocyte repertoire implies that their interaction with the antigen induces a clonal expansion process (positive selection) resulting in the amplification of the number of clones and the production of specific antibodies against the pathogen or the vaccine in biologically relevant quantities. During T-dependent clonal selection, antigen-specific B cell clones undergo somatic hypermutation (SHM) in the V region, allowing the selection and differentiation of high affinity memory B cells, which are the biological basis of vaccination. Therefore, in order to develop a universal influenza vaccine [18], it is essential to understand how viral diversity shapes B cell clonal selection and competition to favor or disfavor broadly neutralizing B cell clone selection.

Within the context of the recent 2009 influenza pandemic, it was surprising that the virus belonged to the A(H1N1) subtype due to the expectation of a predominance of homotypic neutralizing immunity at population level resulting from natural infection and/or vaccination with pre-pandemic H1N1 strains [19, 20]. However, crystallographic studies of the HA of the new 2009 pdmH1N1 virus revealed its structural and antigenic similarity with the H1N1 virus of the 1918 pandemic, which ceased to circulate among human populations since the 1950s [21].

In spite of having HA of the H1 subtype, the H1 globular domain of the 2009 pdmH1N1 and seasonal pre-pandemic H1N1 viruses have diverged significantly through drift, and therefore were considerably structurally different [21, 22]. Studies in natural infection and vaccination with the 2009 pdmH1N1 strain revealed a predominant heterotypic responses directed to the HA stem, suggesting B cell selection of subdominant clones against conserved epitopes of the HA stem [23, 24]. Thus, the 2009 pandemic was a historic opportunity to longitudinally analyze the immune response in humans against a new viral strain with a high divergence in the H1 globular domain, but conserved stem structure.

In this work, we analyze the induction of homotypic and heterotypic antibody responses to vaccination with pre-pandemic 2008/2009 trivalent Influenza vaccine (08/09 TIV), followed by the 2009 monovalent influenza vaccine (09 MIV). Additionally, the peripheral blood IgG V_H_ B cell repertoire was characterized in six individuals (born after 1950), 7 days after immunization with the 08/09 TIV, when antigen-specific plasmablast mobilization takes place [23, 25]. This repertoire was compared to that induced by a subsequent immunization with the monovalent inactivated vaccine against 09 MIV, and a third vaccination trial with the 2014/2015 TIV, which includes the 2009 pdmH1N1 HA antigen. The comparative analysis of the repertoires in the same individuals allowed the identification of common IGHV usage and signatures and somatic hypermutation pattern associated with seasonal vaccines (08/09 TIV and 2014/15 TIV) in contrast with 09 MIV.

## Methods

### Ethics statement

This study was conducted according to the principles expressed in the Declaration of Helsinki. The study was approved by the Research, Ethics, and Biosafety Committees of the Instituto Nacional de Salud Pública (INSP) (CI: 971), and Instituto de Diagnóstico y Referencia Epidemiológicos (InDRE). Written informed consent of all participants was obtained.

### Vaccination of volunteers and sample collection

**Fig. 1:**
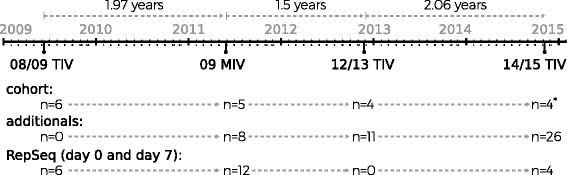
Experimental design timeline of immunizations and sequencing experiments. Six individuals naïve for 2009 pdmH1N1 were vaccinated with the 08/09 TIV and their peripheral blood IgG B cell repertoire was sequenced before vaccination (day 0) and 7 days after vaccination. Two years later, the IgG B cell repertoire of five to six individuals previously vaccinated plus eight additional participants vaccinated with 09 MIV (of 18 participants) were sequenced (day 0 and day 7). Finally, four of the same five participants vaccinated with 08/09 TIV and 09 MIV were vaccinated with 12/13 TIV. The same four participants were then vaccinated with 14/15 TIV and subjected to Rep-Seq (*), as in the previous trials. The overall experiment length from the first to the last vaccination trial was 5 years, 5 months

During the initial phases of the 2009 pdmH1N1 influenza pandemic, six clinically healthy participants aged over 28 and under 41 years working at INSP manifested their intention to receive the 2008/09 trivalent inactivated influenza vaccine (TIV) (FLUARIX, GSK. A/Brisbane/59/2007(H1N1), A/Brisbane/10/2007(H3N2), B/Florida/4/2006). These individuals were invited (after signing an informed consent document) to donate four peripheral blood samples: one before the vaccination (day 0), and the others on days 7, 14, and 30 after the vaccination. Twenty-four months later, five of these individuals and 18 individuals who had not been vaccinated in the 2009 trial and whose vaccination history and exposure to the influenza virus was unknown, were recruited for a second trial to receive the 2009 pdmH1N1 MIV (non-adjuvanted, 15 μg hemagglutinin (HA) of influenza A/California/07/2009 (H1N1) v–like virus Sanofi Pasteur). They donated peripheral blood samples before (day 0), 7, 14, and 30 after the vaccination. Peripheral blood samples for serum and RNA (PAXgene Blood RNA Tubes. PreAnalytiX) were collected from each of the participants through venipuncture, and the total RNA was extracted according to manufacturer’s instructions. Finally, four individuals previously vaccinated with 08/09 TIV and 2009 pdmH1N1 MIV were vaccinated with 2012/13 TIV and 2014/15 TIV (FLUARIX, GSK. A/Christchurch/16/2010 NIB-74XP (H1N1) (an A/California/7/2009-like virus), 273 A/Texas/50/2012 NYMC X-223A (H3N2), and B/Massachusetts/2/2012 NYMC BX-51B), as a part of the Institutional seasonal influenza vaccination program. Blood samples from these individuals were taken as described for the previous vaccination trials. The overall design of the experiment is depicted in Fig. 1.

### Hemagglutination inhibition assays

Hemagglutination inhibition assays (HIA) [26] were performed in order to determine serum antibody titers against the pre-pandemic 2008/2009 A(H1N1) and H3N2 and 2009 pdmH1N1 virus strains in samples of day 0 and 30 days after the vaccination with 08/09 TIV and 09 MIV. In short, non-specific agglutinins were eliminated and serial serum dilutions were mixed with an equal amount of PBS with 8 hemagglutinating units of each viral strain. The dilution at which the tests were considered positive was 1:40. Seroconversion to the vaccine was defined as a four-fold increase in the day 0/day 30 post-vaccination titers.

### Plate microneutralization tests

This assay [27] makes it possible to quantify total neutralizing antibodies against the influenza virus, not only those directed against the HA globular domain. For this purpose, serial serum dilutions (days 0, 7, 15, and 30) were incubated with viral strains A(H1N1) 2008, A(H3N2) 2008 and AH1N1pdm 2009), and the residual virus-serum mix infectiveness for MDCK of cells was determined using an ELISA with an anti-NP antibody. Neutralizing titers were defined as the reciprocal of the highest serum dilution that totally neutralized the viral infection. The minimum dilution at which a test was considered positive was 1:80. Seroconversion to the vaccine was defined as a four-fold increase in the day 0/day 30 post-vaccination titers.

### Generation of V_H_ gene libraries for massive cDNA sequencing

Peripheral blood RNA was stored at -70 °C, and an aliquot was used to analyze its concentration and integrity through capillary electrophoresis in a 2100 BioAnalyzer, with the Agilent RNA 6000 Pico kit (Agilent). To characterize the clonal structure of the B cell repertoire in response to 08/09 TIV, 09 MIV, and 14/15 TIV, and to avoid potential amplification biases [28], we used 5′RACE-PCR [29]. cDNA was generated for the V_H_ region of the IgG compartment on day 0 (pre-immune) and 7 days after the vaccination, to coincide with the peak of circulating antigen-specific plasmablasts [23, 25]. For each V_H_ amplicon library, 1 μg of RNA was used to synthesize cDNA. The cDNA generated had a known DNA sequence (adaptor) at the 3′ end, and the oligonucleotide (dT) sequence at the 5′ end. cDNA was used as template to produce amplicons containing the diversity generated through V(D)J recombination of the heavy chain. We substituted the TS-PCR oligonucleotide of the Matz protocol [29] for the FpAmpTA oligonucleotide, which is identical to TS-PCR except that it contains the sequence of the A adaptor, which is necessary for the massive amplicon sequencing protocol developed by 454-Roche. The gene libraries were generated using FpAmpTA together with the TBIgGHu oligonucleotide, which primes amplification specifically in the segment that encodes for exon I of IGHG (1-4) and contains adaptor B sequence required for massive sequencing. The products of the 5′-RACE-PCR reactions (500-600 bp) were analyzed by electrophoresis in agarose gels at 1.5 % and were purified from the gel using the MiniElute PCR purification kit (Qiagen). The concentration and integrity of the gene libraries were analyzed through capillary electrophoresis in the 2100 BioAnalyzer, using the High Sensitivity DNA kit (Agilent).

### High throughput DNA sequencing of gene libraries

Approximately 100 ng of each of the 44 gene libraries were analyzed for clonal amplification by emulsion PCR using the 454-Roche GS emPCR kit, according to the manufacturer’s instructions. The sequencing of the gene libraries was carried out using the GS FLX Titanium Sequencing kit XLR70, according to the manufacturer’s instructions. This system allows the generation of sequences with an average length of 450 bp. In order to sequence at least the CDRH3 region and partially the IGHV region, the sequencing was carried out in antisense, that is, from the B adapter. Raw sequencing files were submitted to NCBI-SRA: BioProject ID: PRJNA301150; Accessions: SAMN04240435-78.

### Bioinformatics analysis with the ImmunediveRsity platform

We have developed ImmunediveRsity [30], a bioinformatics analysis platform based on the R language for the automated analysis of the structural diversity of the B-lymphocyte repertoire. This data processing platform begins with quality screening. Sequence files were screened in order to exclude non-V_H_ sequences (for example, germ-line transcripts), by mapping the human genome, reads under 200 bp and reads with an average quality value of < Q28. After quality filtering, ImmunediveRsity assigns IGHV and IGHJ segment use to each read using IgBLAST (http://www.ncbi.nlm.nih.gov/igblast/). Incorrect assignment of the IGHD segment is very common (approximately 50 % of the cases); therefore, the IGHD assignment is omitted. For every different V-J rearrangement, each read is assigned to a V_H_ clonotype through identification of the segment encoding for CDRH3 using hidden Markov models [31], followed by its recursive clustering with USEARCH [32], based on length identity and a 97 % sequence identity. Because only the heavy chain variable region (IGHV) was sequenced, this method does not describe *sensu stricto* lineages (clonally related IGH + IGL pairs). Henceforth, we describe IGHV *sensu lato* lineages as the consensus generated through the recursive clustering of reads with ≥99.5 % identity that belong to the same V_H_ clonotype, but diversified by somatic hypermutation. ImmunediveRsity output files for each sequenced library can be found at http://201.131.57.23:8080/influenza-project/.

### Somatic hypermutation analysis

For each lineage consensus, the numbers of non-synonymous and synonymous mutations were obtained with IMGT/HighVQuest [33]. Only productive lineages were used for random sub-sampling (670 lineages per library, which corresponds to the library with the least number of lineages). The proportion of mutations (pM-VH) was calculated as the percentage of total of mutations in V_H_ region, excluding the CDRH3, divided by its length. To avoid non-independence effects from lineages derived from large clonotypes, SHM was also calculated in the largest lineage per clonotype from 250 randomly sampled clonotypes.

### Analysis of the structural diversity of the B-lymphocyte repertoire

In order to quantify the clonal and lineage diversity, rarefaction curves [34] were plotted with growing subsamples of 1,000 reads. The Shannon-Weaver index (***D***^***SW***^) (entropy) was used as a *proxy* to clonotype and lineage diversity [35]. For IGHV segment use analysis, differences between the pre-immune groups and at 7 days post-vaccination groups were statistically evaluated using the GraphPad Prism 5.04 software. To identify the changes induced by the corresponding vaccines, the relative frequency of unclustered reads and V_H_ clonotypes of the post-vaccination use of IGHV segments was subtracted from the corresponding pre-immune frequency (Δ day 7 – day 0).

### Principal component analysis of diversity, IGHV use, and mutation frequency

Comparisons among 2008/09 TIV, 09 MIV and 2014/15 TIV based on rarefaction analysis (d7/d0 ratio at 12,000 read sampling effort), Shannon entropy (***D***^***SW***^ d7/ ***D***^***SW***^ d0 ratio), *IGHV1-69*, *IGHV3-7*, and *IGHV4-39* use (Δ day 7 – day 0) and mutation rate at day 0 and at day 7 post vaccination for each individual were performed with a multilevel principal component analysis (PCA) [36]. Graphical representation of Component 1 (PC1) versus Component 2 (PC2), selected by the criteria of proportion of explained variance (that is, accounts for as much of the variability in the data as possible) was done with a biplot graph. This analysis was conducted using R software [37] and FactoMineR library.

### Clonal expansion analysis and recombinant monoclonal antibody production

Clonal expansion using particular IGHV segments were defined as an increase of ≥3 standard deviations (SD) in the change of clonal frequency (Δ day 7 – day 0). The two biggest lineages of the largest heavy chain clonotypes of clonal expansions observed *in silico* were selected for experimental validation of anti-Influenza virus specificity. The sequence corresponding to the V_H_ region flanked by *EcoR*I and *Nhe*I restriction sites were synthesized as gene fragments (Gblocks, IDT) and cloned in the expression vector of human antibody heavy chains pVAJO-CHG1, plasmid coding for human IgG1, as described [38]. The selected V_H_ sequences were matched with seven different variable region sequences of light chain (VL) (See Additional file 1). The V_L_ sequences, synthesized also as gene fragments (Gblocks, IDT), were cloned in the expression vector of antibody light chains pVAJO-CLhk or pVAJO-CLhl. The sequence of the recombinant plasmids was verified by Sanger sequencing. To produce monoclonal antibodies, each heavy chain-encoding plasmid was co-transfected with each of the light chain-encoding plasmid into HEK 293 T cell line (Thermo) and supernatants were collected 48 h later.

### Validation of anti-influenza specificity by ELISA

After transfection, IgG production in supernatants was verified by anti-human IgG ELISA (data not shown). IgG positive supernatants (100 μL) were then tested by ELISA for reactivity against the 09 MIV (influenza A/California/07/2009 (H1N1) v–like virus Sanofi Pasteur) or 08/09 TIV (FLUARIX, GSK. A/Brisbane/59/2007(H1N1), A/Brisbane/10/2007 (H3N2), B/Florida/4/2006) coated-96 well plates in pH 7.4 PBS/Tween-20 0.1 % (300 ng per well). After washing, wells were incubated with goat anti-human IgG coupled with HRP (1:5,000. Abcam), washed, and read at 490 nm.

## Results

### Vaccination with the 08/09 TIV does not induce seroconversion against 2009 pdmH1N1

**Fig. 2:**
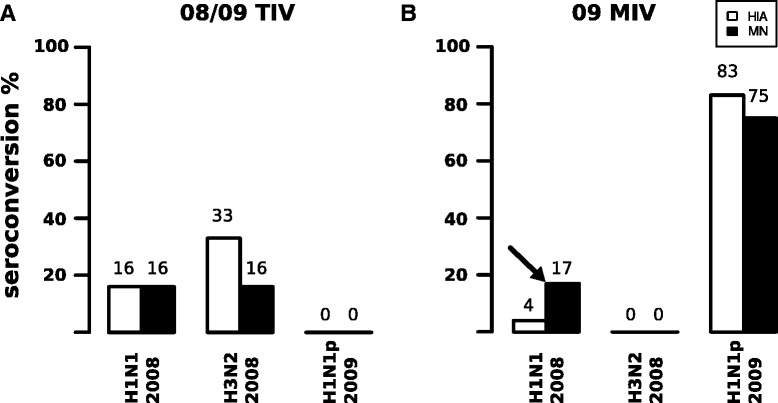
Vaccination with TIV induced homotypic seroconversion (**a**), while 09 MIV induced heterotypic seroconversion against pre-pandemic H1N1 (**b**). Homotypic and heterotypic seroconversion rates for vaccination with TIV and 09 MIV. TIV showed low seroconversion rates for vaccine strains due to pre-exposure (high pre-vaccination titers) (n = 6). The 09 MIV vaccine had a higher homotypic seroconversion (83 % and 75 %) and heterotypic seroconversion against 2008 H1N1 in 17 % of the immunized participants (n = 23) (arrow)

During the onset of the pandemic (May, 2009), six individuals were vaccinated with the 08/09 TIV. The pre-immune serum of three (50 %) and two (33.3 %) individuals showed positive hemagglutination inhibition assay (HIA) for 2008 A(H1N1) and 2008 A(H3N2), respectively, which is indicative of pre-exposure to seasonal viruses and is expected among the general population. All pre-immune samples were negative for 2009 pdmH1N1 in HIA, while 5/6 (83 %) presented low but positive titers (≥80) in the microneutralization test (MN) (Additional file 2). Seroconversion rate as measured by HIA for pre-pandemic H1N1 and H3N2 strains was 16 % and 33 %, respectively. No individual showed seroconversion against the 2009 pdmH1N1 in HIA and MN in response to immunization with TIV (heterotypic seroconversion) (Fig. 2a). The high seropositivity rate to pre-pandemic viruses is consistent with previous exposures to different influenza viruses of the studied population. The absence of heterotypic seroconversion (against 2009 pdmH1N1) is consistent with the promotion of homotypic neutralizing responses by vaccination with TIV.

### Vaccination with 09 MIV induced seroconversion against 2008 A(H1N1) in a subgroup of individuals

Two years after immunization with TIV, five out of the six immunized individuals and an additional group of 18 individuals were immunized with the 09 MIV vaccine against the 2009 pdmH1N1 virus. As in the TIV assay, none of the 23 individuals tested positive for 2009 pdmH1N1 with HIA on day 0; however, 14 individuals (60 %) showed low but positive titers (≥80) in MN. Seropositivity against 2008 A(H1N1) and 2008 A(H3N2) measured with HIA on day 0 was 0 % and 8 %, respectively, whereas measured with MN was 47 % and 65 %, respectively (Additional file 2).

As a result of 09 MIV immunization, the homotypic seroconversion rates for 2009 pdmH1N1 in HIA and MN were 86 % and 75 %, respectively. There was no heterotypic seroconversion for 2008 H3N2 in both tests. Interestingly, 4/23 individuals (17 %) showed heterotypic seroconversion against 2008 A(H1N1) in MN, but not in HIA (Fig. 2b). The apparent inconsistency in the results of seroconversion between MN and HIA may be explained by the induction of a cross-neutralizing antibody response to HA antigenic determinants outside the HA1 domain (that is, anti-stem antibodies). These results indicate that in addition to the expected homotypic response, the 09 MIV vaccine induced in some individuals a heterotypic response, which is consistent with recent findings that 09 MIV re-stimulated B cells that recognize common antigenic determinants between 2008 A(H1N1) and 2009 pdmH1N1 [23, 24, 39].

### Sequencing the peripheral blood B cell repertoire

To structurally characterize and compare the B cell clonal response to vaccination with 08/09 TIV, 09 MIV, and 14/15 TIV, as well as to identify whether there are repertoire signatures associated to each vaccine and to the homotypic and heterotypic responses, we sequenced the V_H_ region of IgG from total RNA of peripheral blood lymphocytes before and 7 days after vaccination in each vaccination trial. We generated a total of 778 mbp and 1.7 million sequencing reads, with an average of 17.6 mbp and 40,000 reads per V_H_ amplicon library. The average read length was 449 ± 51 bp, which is sufficient to cover the entire V_H_ region length. This allowed a detailed analysis of 218,910 lineages (unique heavy chain, see Materials and Methods), corresponding to an average of 4,975 V_H_ lineages per V_H_ amplicon library (Table 1). See Additional file 3 for detailed repertoire metrics.

**Table 1:** Sequencing metrics of the analyzed V_H_ sequences

Sequencing results	Parameter	Totals^a^	Amplicon library (Average ± SD)^b^
Raw output	454 reads	1,792,504	40,738 ± 27,496
	Total bases (bp)	778,487,667	17,692,901 ± 12,073,169
	Average length (bp)	ND	483 ± 77
Quality and size filters	Eliminated reads (%)	27 %	24 % ± 14
Sequences included in the analysis	Analyzed with ImmunediveRsity	1,302,865	29,610 ± 18,114
	Clonotypes	71,568	1,626 ± 938
	Lineages	218,910	4,975 ± 3,378

^a^Total parameters of five 454-Roche Titanium sequencing runs. Forty-four gene libraries were generated from 13 individuals

^b^Each gene library corresponds to one of six conditions: day 0 and day 7 after vaccination with TIV2008/2009, day 0 and day 7 after vaccination with 09 MIV2009, and day 0 and day 7 after vaccination with TIV2014/2015

### Vaccination with TIV and 09 MIV induced different responses in terms of the clonotypic and lineage diversity of the IgG peripheral blood B cell repertoire

**Fig. 3:**
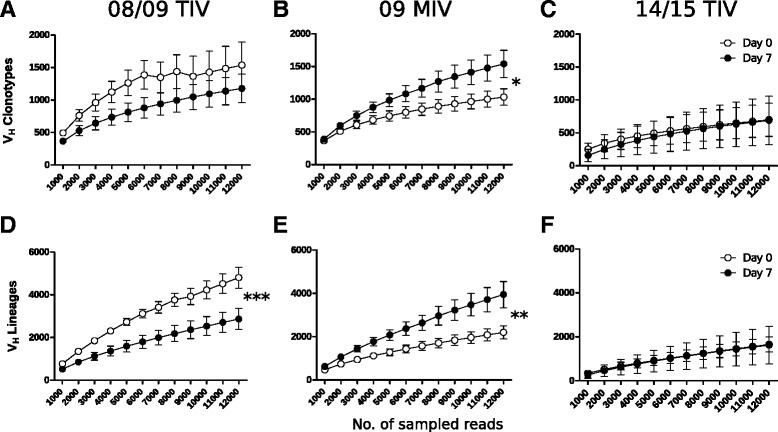
Vaccination with 08/09 TIV induced a reduction of the clonal and lineage diversity, while 09 MIV induced an increase in the diversity. A rarefaction analysis (**a**-**d**) was performed with progressive subsamples of 1,000 reads (axis *x*), plotted against the average standard error of clonal groups (**a**-**c**) and lineages (**d**-**f**) for 08/09 TIV (**a** and **d**), 09 MIV (**b**, **e**), and 14/15 TIV (**c**, **f**) vaccination trials. The empty symbols represent the values for day 0 (pre-immune), while the black symbols stand for those for day 7 after vaccination (two-way ANOVA. *P <0.05; **P <0.01; ***P <0.001). Fluctuations above 7,000 reads in pre-immmune are due to insufficient sequencing depth in two individuals (see also Additional file 3)

Antigen recognition in the secondary lymphoid organs involves clonal selection and diversification by SHM, which affects the clonal structure and diversity of the repertoire. To quantify the peripheral B cell clonal diversity and its modification in response to vaccination, we used in population ecology techniques to assess species richness and diversity. We expected that vaccine induced mobilization of clonally related plasmablasts displaying high levels of Ig transcription, compared to memory B cells would manifest as a rapidly saturating rarefaction curve. Indeed, as shown in Fig. 3, 08/09 TIV induced a reduction of clonotype and lineage diversity compared to the pre-immune sample (Fig. 3a and d). Conversely, vaccination with 09 MIV was associated with an increase in clonal group and lineage diversity post-vaccination (Fig. 3b and e). Interestingly, immunization with 14/15 TIV showed no differences between the pre-immune and 7 days post immunization (Fig. 3c and f).

To confirm the results of the rarefaction analysis, we estimated the Shannon-Weaver diversity index (*D*^*SW*^) on days 0 and 7 after vaccination in each trial (08/09 TIV, 09 MIV, and 14/15 TIV). To account for differences in the number of circulating IgG+ B cells within individuals in the different vaccination trials, we calculated the *D*^*sw*^ day 7/ *D*^*sw*^ day 0 ratio. The 08/09 TIV trial consistently showed ratios <1.0 (reduction of the diversity with respect to day 0) in both clonal groups and lineages (Additional file 4, A and B), indicating a reduction of clonal and lineage diversity. Contrastingly, the *D*^*SW*^ day 7/ *D*^*SW*^ day 0 ratio in response to vaccination with 09 MIV was consistently higher than with both TIVs and above 1.0 (Additional file 4, A and B). As for the 08/09 TIV trial, the *D*^*sw*^ day 7/ *D*^*sw*^ day 0 ratio in response to a third vaccination with 14/15 TIV was below 1.0. These results suggest that the clonal response to 08/09 and 15/15 TIV is more similar compared to 09 MIV and suggests that vaccination with 09 MIV promotes mobilization of a more diverse plasmablast population into peripheral blood.

### Immunization with TIV and 09 MIV induced differential changes in the clonal frequency of B cells using *IGHV1-69*, *IGHV3-7*, and *IGHV4-39*

**Fig. 4:**
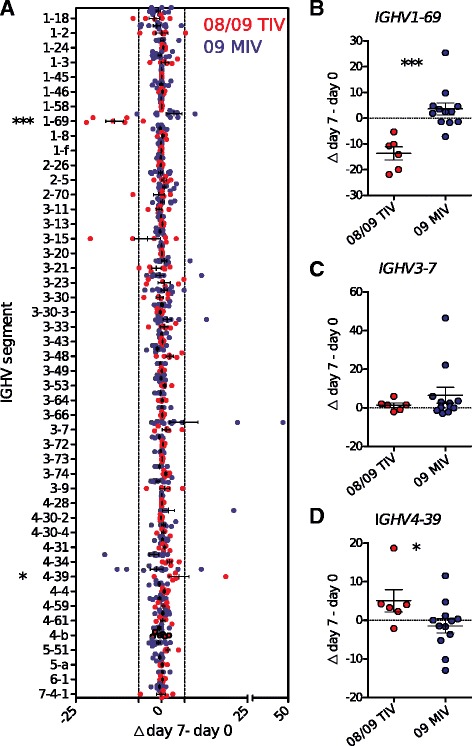
Effect of immunization with TIV and 09 MIV on the repertoire of peripheral B-lymphocytes. The change in the relative frequency of IGHV segment use (Δ day 7 – day 0) for TIV (red dots, n = 6) and with 09 MIV (blue dots, n = 12), for 47 IGHV segments (**a**), and, in detail, for *IGHV1-69* (**b**), for *IGHV3-7* (**c**), and for *IGHV4-39* (**d**). The dotted lines represent ± 2 standard deviations (±6.7) from the mean (0) (Mann–Whitney test. ***P <0.001, *P <0.05)

To characterize the B cell response to each vaccine in terms of the BCR structural determinants involved in recognition of common and unique antigenic determinants of the different influenza virus strains, we determined the relative frequency usage of IGHV segments as relative transcription (unclustered reads), or as their proportional usage frequency at the clonotypic level. Changes in the frequency usage were expressed as the difference between post-immunization (day 7) minus pre-immune frequencies (Δ day 7 – day 0). For the 2008/09 TIV, only *IGHV1-69* and *IGHV4-39* out of 47 IGHV segments analyzed, showed significant differences at the clonotypic level in response to immunization with either of the two vaccines (Fig. 4a) (two-way ANOVA. *P* <0.0001 and *P* <0.001, respectively).

In most of the analyzed individuals, the *IGHV1-69* clonal frequency was the opposite according to the vaccination assay: in response to 08/09 TIV, the post-vaccination clonal frequency was lower than pre-vaccination levels in all individuals. Conversely, the post-vaccination clonal frequency of *IGHV1-69* was equal or higher than the pre-immune level in response to 09 MIV (Fig. 4a and b). As for B cells using *IGHV4-39*, a similar but opposite behavior was observed, since TIV induced a clonal frequency increase, whereas 09 MIV induced a reduction (Fig. 4a and d).

Clonal frequency of B cells using *IGHV3-7* increased significantly only in response to vaccination with 09 MIV (*P* <0.0001). This increase occurred due to two outlier individuals (i05 and i07), in which a high fraction of clones used *IGHV3-7* (from 3 % at day 0 to 49.5 % and 3.7 at day 0 to 26 %, respectively) (Fig. 4a and c), occupying a large fraction of the V_H_ transcriptome (from 2 % at day 0 to 81 % and 1.7 % at day 0 to 57 %, respectively, Fig. 4c). To identify if allelic differences could favor the selection of some clonotypes, we identified that individuals i05 and i07 that showed clonal expansions in *IGHV3-7* were heterozygous for IGHV3-7*01/IGHV3-7*03 and homozygous for IGHV3-7*01, respectively. The rest of the individuals that did not show clonal expansion in *IGHV3-7* expressed were homozygous for *01 (4/13), heterozygous *01/*03 (5/13) or heterozygous for *03 (2/13). In the heterozygous individual (i05), the expanded clones corresponded to the *03 allele. Thus, at least for the *IGHV3-7* segments, alleles *01 and *03 do not determine a selective clonal advantage for 09 MVI. Taken together, these results suggest that different individuals respond similarly in terms of *IGHV1-69* and *IGHV-4-39* segment use, in which the clonal structure of the peripheral blood B cells was modified inversely in response to both vaccines. Furthermore, the *IGHV3-7* segment was markedly expanded in two individuals only in response to 09 MIV.

### Re-vaccination trial with the 2014/1015 TIV recapitulates the 2008/2009 pre-pandemic TIV vaccination in terms of *IGHV1-69* and *IGHV4-39* usage

**Fig. 5:**
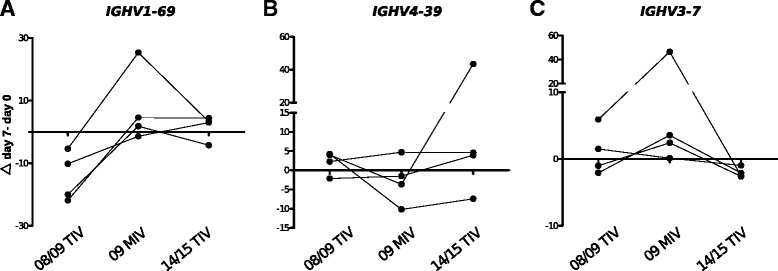
Longitudinal changes in IGHV segment usage upon influenza vaccination. The change in the relative V_H_ clonotype frequency of IGHV segment use (Δ day 7 – day 0) in the same four individuals vaccinated with 08/09 TIV, 09 MIV, and 14/15 TIV for (**a**) *IGHV1-69*, (**b**) *IGHV4-39*, and (**c**) *IGHV3-7*

The increased use of *IGHV1-69* in response to 09 MIV has been implicated in the predominant anti-HA2 heterotypic response resulting of natural infection and vaccination with the 2009 pdmH1N1 [23, 24, 39]. We asked if repeated exposure after an initial challenge with 2009 pdmH1N1 would revert the pattern of IGHV use to that observed with the 08/09 TIV. Thus, we evaluated relative IGHV use at the clonotype level 7 days post vaccination with 14/15 TIV in the same four individuals vaccinated with 08/09 TIV, followed by 09 MIV and 2011-12 TIV (Fig. 1). We observed a tendency to a reduction in the usage frequency of *IGHV1-69* and *IGHV3-7* (Fig. 5a and b). Likewise, *IGHV4-39* showed a tendency to increase (Fig. 5c).

**Fig. 6:**
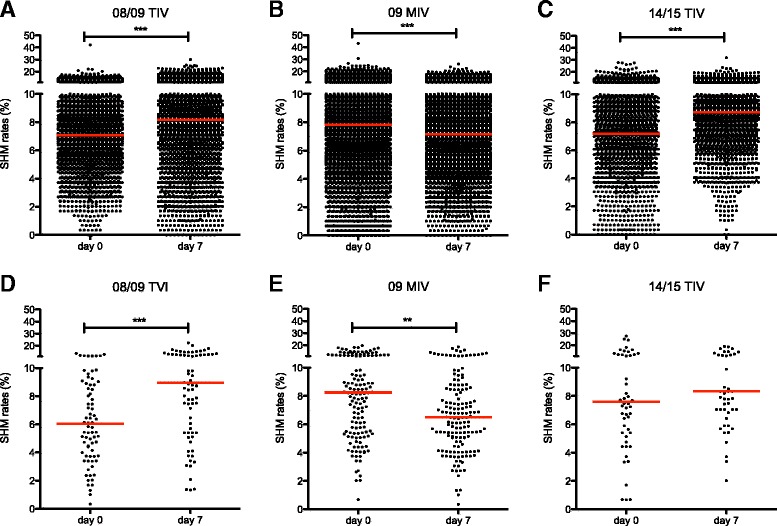
Somatic hypermutation analysis in response to 08/09 TIV, 09 MIV, and 14/15 TIV. SHM rates (% of mutations in V_H_ region) are shown according to vaccination trial. In the first approach, 670 randomly sampled lineages per individual were selected and plotted (**a**-**c**). (**a**) 08/09 TIV; (**b**) 09 MIV; and (**c**) 14/15 TIV. A second sampling approach was used based on selecting the single largest lineage of 250 V_H_ clonotypes (**d**-**f**) (Kruskal–Wallis test. Dunn’s correction for multiple testing. **P <0.01, ***P <0.001)

A preferential use of certain IGHV segments and low rates of somatic hypermutation have been described in the primary antiviral response in mouse and in human models [40, 41]. Thus, we assessed the overall SHM rate in the same four individuals before and 7 days post vaccination with 08/09 TIV, followed by 09 MIV and 14/15 TIV. Increased SHM was observed 7 days post vaccination with 08/09 TIV (Fig. 6a). Contrastingly, SHM rates were reduced 7 days post vaccination with 09 MIV (Fig. 6b). Nevertheless, vaccination with 14/15 TIV caused SHM rates to return to the pattern observed with the 08/09 TIV (Fig. 6c). It is possible that random sampling of lineages, particularly in those large clonotypes composed by many lineages could cause non-independence effects that could compromise SHM accuracy. Thus, we also estimated SHM by sampling one lineage per randomly sampled clonotype. Consistent with the previous strategy, SHM were reduced post vaccination with MIV and increased post vaccination with 08/09 TIV and 14/15 TIV (Fig. 6d-f). Moreover, somatic hypermutation could affect differentially according to IGHV use, so we estimated SHM in expanded (*IGHV1-69*, *IGHV3-7*, and *IGHV4-39*) or non-expanded lineages using particular IGHV segments (*IGHV1-3*, *IGHV3-15*, and *IGHV4-59*). In the case of *IGHV1-69*, SHM rates were identical to the pattern observed for the ‘bulk’ analysis. For *IGHV3-7*, no significant differences were found. For *IGHV4-39*, an increase in SHM post-vaccination was significant only for TIV 08/09 (Additional file 5).

**Fig. 7:**
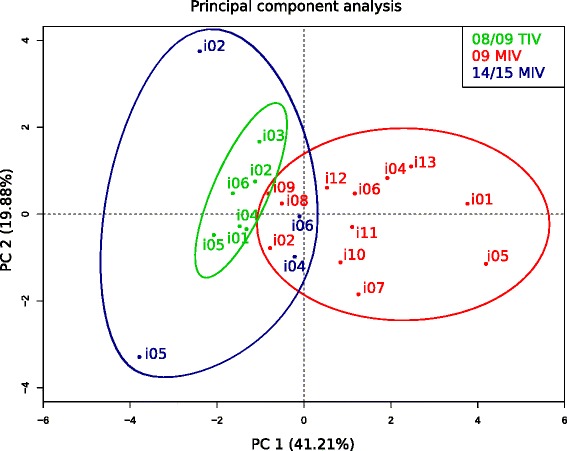
Principal component analysis of influenza vaccinees according to each trial. Principal Component biplot showing clustering of vaccinees according to both TIVs or 09 MIV immunization. PCA was constructed using results of clonotype and lineage rarefaction and entropy analysis, *IGHV1-69*, *IGHV3-7*, and *IGHV4-39* use and SHM rates per individual and trial. 08/09 TIV (green), 09 MIV (red), and 14/15 TIV (blue)

Taking together the results of clonotypic and lineage diversity, the change of IGHV use upon vaccination and the differences in the mutation rates indicate that immunization with the new virus induced a different clonal response pattern than re-immunization with seasonal variants. To prove so, a multilevel PCA was performed to search for association patterns between diversity, IGHV use, SHM rates, and vaccine type. We used the day 7/day 0 ratio of clonotype and lineages species, *D*^*sw*^ day 7/ *D*^*sw*^ day 0 ratio, the change of IGHV clonotype frequency (Δ day 7 – day 0) and the mean proportion (%) of all mutations, as variables for the analysis. Two components, PC1 and PC2, explained 41.2 % and 19.8 % of the variance, respectively, with a cumulative proportion of 61 %. Biplots of PC1 and PC2 showed two major clusters, one containing the majority of the TIV vaccinations regardless of their pre-pandemic or post-pandemic status, and the second cluster containing the 09 MIV (Fig. 7). Thus, this non-supervised approach robustly support that repeated seasonal exposures elicit common clonal selection patterns that differ from those elicited by an exposure to a new variant.

### IGHV usage signatures associated with heterotypic seroconversion include other segments than *IGHV1-69*

**Fig. 8:**
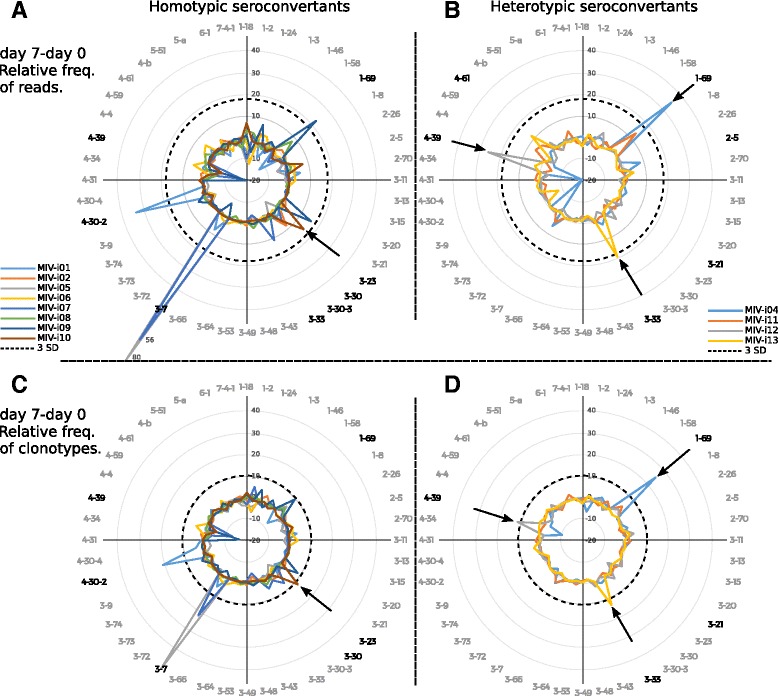
Effect of immunization with 09 MIV on the repertoire of peripheral B-lymphocytes with homo- and heterosubtypic seroconversion. Changes in the relative frequency of use of IGHV segments (Δ day 7 – day 0) for individuals with homotypic seroconversion (n = 7) (**a**, **c**), and with heterosubtypic seroconversion (n = 4) (**b**, **d**) for 47 IGHV segments. IGHV usage is expressed as relative transcription (unclustered reads) (**a**, **b**), and as the proportion of clonotypes using a particular IGHV segment (**c**, **d**). The dotted line indicates three standard deviations, which corresponds to 18.5 for relative transcription and 10.3 for clonotypic frequency above the means, 0.0014 and 0.003, respectively. Arrows indicate clonotypes selected for experimental validation. *IGHV* segments in bold letters indicate relevant expansions

The observed changes in *IGHV1-69* at day 7 post-08/09 TIV and 09 MIV immunization (Figs. 4b and 5a) and the implication of this segment in broad influenza virus strain neutralization [18] suggested that the four individuals that developed heterotypic seroconversion upon 09 MIV immunization would display *IGHV1-69* clonal expansions. Thus, we split the 09 MIV trial in two groups according to their heterotypic and homotypic seroconversion response. Only one individual (i04) with positive heterotypic seroconversion had clonal expansion of the *IGHV1-69* (>3 standard deviations) (Fig. 8a and c). Another individual (i11) also had positive heterotypic seroconversion and an expansion in *IGHV1-69*, however did not reach >3 standard deviations selection threshold. The remaining two individuals with heterotypic seroconversion showed individual expansions of segments *IGHV4-39* (i12) and *IGHV3-33* (i13), respectively (Fig. 8a and c). As for the individuals who did not undergo heterotypic seroconversion, segment *IGHV1-69* was expanded in two out of seven individuals (Fig. 8b and d). Two individuals (i10 and i01) also showed expansions in segments *IGHV3-23* and *IGHV4-30-2*, respectively (Fig. 8a and c, Additional file 6). The Phe60 in the CDRH2 of IGHV1-69 has been implicated in the neutralizing activity of some anti-HA stem antibodies [18, 42]. We did not find any correlation between the presence of Phe60 and clonal expansion nor heterosubtypic seroconversion (Additional file 7). Taken together, these results suggest that *IGHV1-69* clonal expansions do not correlate exclusively with the heterotypic response, and allowed us to identify IGHV segments other than *IGHV1-69* that may be involved in the heterotypic seroconversion.

Another hypothesis that could be tested under our experimental design is the original antigenic sin (OAS) hypothesis, which states that subsequent antibody responses to new viral strains are dominated by antibodies with higher affinity for the original immunizing variant [43, 44]. We searched for identical V_H_ clonotypes shared by the same individual at day 7 post vaccination with 08/09 TIV, 09 MIV, and 14/15 TIV (n = 4 individuals; four possible comparisons: 08/09 TIV vs. 09 MVI, 08/09 TIV vs. 14/15 TIV, 09 MVI vs. 14/15 TIV, and 08/09 TVI vs. 09 MVI vs. 14/15 TIV). The average number of clonotypes per individual in the three vaccination trials was 1,894 ± 1,255. The absolute number of clonotypes analyzed for each individual are in Additional file 3. No shared clonotypes were found common to the three vaccination trials in any of the individuals. Only four clonotypes in individual i04 were shared at day 7 post vaccination between 08/09 TIV and 09 MVI. Individual i04 presented heterotypic seroconversion upon 09 MIV immunization and two of the shared clonotypes used *IGHV1-69* and contributed to the *IGHV1-69* clonal expansion observed in this individual (Fig. 8b and d). This finding is consistent with the new 2009 pdmH1N1 virus selecting B cells specific for shared epitopes with the pre-pandemic virus. However, we found no evidence supporting OAS.

**Fig. 9:**
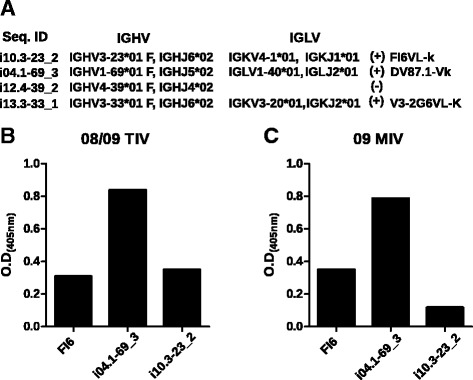
B cell clonal expansions associated with heterotypic seroconversion are influenza-specific. (**a**) Structural features of recombinant monoclonal antibodies in terms of VDJ segment use and successful VL pair. (**b**) Enzyme-linked immunosorbent assay of three recombinant monoclonal antibodies derived from *in silico* repertoire mining of clonal expansions in segments *IGHV1-69 and IGHV3-23 against TIV antigens, and* (**c**) *against MIV antigen.*

To prove that the observed clonal expansions associated with heterotypic and homotypic seroconversion were influenza virus-specific, we selected the largest clone of *IGHV1-69* (i04), *IGHV3-33* (i13), and *IGHV4-39* (i12) (heterotypic seroconvertants), and *IGHV3-23* (i10, homotypic seroconvertant) clonal expansions to produce the corresponding recombinant monoclonal antibody. In the case of IGHV1-69, the clonotype shared at day 7 post immunization with 08/09 TIV and 09 MIV was selected (i04.1-69_3). As no information regarding the *in vivo* corresponding IgL pair, IgL genes derived of published mAbs were used for co-transfection (Additional file 1). The characteristics of the recombinant mAbs are described in Fig. 9a. The *IGHV1-69*, *IGHV3-33*, and *IGHV3-23* antibodies were positive against 09 MIV by ELISA. The i04.1-69_3 mAb (IGHV1-69) reacted with both 08/09 TIV and 09 MIV and gave stronger signals than the pan-influenza FI6 mAb [45]. The *IGHV3-23* reacted against 08/09 TIV with higher relative affinity than to 09 MIV (Fig. 9b and c). No reactivity against 09 MIV was found for *IGHV4-39* paired with any of the light chains tested (Fig. 9).

## Discussion

Through the combination of traditional serological analysis, high throughput sequencing applied to the analysis of the diversity of the repertoire of B cells and bioinformatics analysis, we have studied the clonal response to the 2008/2009 trivalent influenza vaccine, as well as to a subsequent vaccination with the 2009 pdmH1N1 influenza vaccine and the 2014/2015 trivalent influenza vaccine.

Previous work aiming at the characterization of the B cell repertoire in response to influenza vaccination using high-throughput sequencing revealed the power of this approach in understanding clonal structure, mutation patterns, the influence of age, and structural convergence [46–49]. In this work, we compared in the same four individuals, B cell clonal responses to seasonal pre-pandemic 08/09 TIV, pandemic 09 MIV, and seasonal post-pandemic 14/15 TIV applied during a 5-year period. Since the study was initiated right at the seasonal to pandemic transition (May, 2009), we could be certain that the initial group of TIV vaccinees were naïve for the 2009 pdmH1N1 virus, providing a unique opportunity to define a B cell repertoire baseline to a new virus, as a reference to analyze the B cell clonal response to further re-infections (or vaccination) with antigenic drift variants of the pandemic virus, as it becomes seasonal.

The comparative analysis of the serological response to the 08/09 TIV and 09 MIV vaccines made it possible to identify heterotypic neutralizing responses in four out of 23 individuals vaccinated with 09 MIV, evaluated as a four-fold increase of the neutralizing titers against a heterologous virus (not present in 09 MIV). This is in agreement with previous analyses of the response to 09 MIV or in individuals with a natural infection with the 2009 pdmH1N1 virus, which demonstrated that the response was mainly cross-reactive and cross neutralizing [23, 24, 39]. The induction of heterotypic neutralizing responses against new viruses such as the 2009 pdmH1N1 is relevant, but these are usually of low intensity in response to vaccination with seasonal variants [50], as was confirmed in this study (Fig. 1, Additional file 2).

By means of the bioinformatics analysis of the sequences of the V_H_ region and the application of analytical and statistical methods oriented to filter the ‘noise’ (inherent to the repertoire in individuals with a history of exposure to different antigens), we identified consistent response patterns between both TIV (pre and post-pandemic) vaccines, that were clearly different to the pattern elicited by 09 MIV. In order to estimate the diversity and heterogeneity, we applied approaches derived from population ecology to the study of the ‘lymphoid micro-ecosystem’ [34, 35]. Both, the pre-pandemic 2008/09 and the post-pandemic 2014/15 vaccines induced a reduction of the post-vaccination clonal and lineage entropy with respect to its pre-immune value, which could be explained by the numeric expansion and selection of a reduced group of high Ig-expressing plasmablasts (Fig. 3 and Additional file 4). Similar results were obtained for the TIV by measuring the clonality index derived by multiple sampling [49, 51]. An important finding of this work, supported by the rarefaction analysis and estimates of the *D*^*SW*^ indices, is the paradoxical increase in the clonal and lineage diversities in the post-vaccination repertoire induced by the 09 MIV, suggesting a highly diverse clonal mobilization of B cell clones toward the peripheral blood as consequence of the exposure to the new virus, for which memory B cell responses are lacking. Interestingly, in a third vaccination with 14/15 TIV there was no difference between pre and post-immunization rarefaction curves (Fig. 3). Moreover, the *D*^*SW*^ indices in 14/15 TIV resembled that of the 08/09 TIV (Additional file 4), suggesting that the clonal response to repeated seasonal immunizations with TIV tends to return to the pre-pandemic pattern.

The frequency of *IGHV1-69* segment use was reduced after vaccination with TIV (Fig. 4a and b). This is noteworthy, because this segment has been described in heterotypic neutralizing antibodies that bind the HA stem of various influenza subtypes, independently of the light chain (VL) and CDRH3. This atypical recognition depends, at least partly, upon the presence of the CDRH2 encoded Phe60 in the germ-line *IGHV1-69* segment, allowing essential hydrophobic interactions with the region of the HA stem, which takes part in the fusion of the membranes during the infection process, and is highly conserved in various influenza virus subtypes [10, 18, 24, 42, 52, 53].

The reduction in the use of *IGHV1-69* in response to TIV and the lack of shared *IGHV1-69* clonotypes after 09 MIV vaccination (Fig. 4a and b) could imply that the *IGHV1-69* expressing B cells are displaced by clonal competition with clonotypes directed against the immunodominant epitopes of the HA globular domain. Accordingly, we observed that TIV promoted the expansion of the use of *IGHV4-39*, a segment involved in the recognition of the HA globular domain and with homotypic neutralization capacity [23, 24] (Fig. 4a and d). Further repertoire analysis in four individuals exposed to two post-pandemic TIV (2012-13 and 2014-15) revealed a trend of *IGHV1-69* and *IGHV4-39* usage towards the pre-pandemic pattern. Because the globular domain of 2009 pdmH1N1 is very different from that of pre-pandemic H1N1 strains (approximately 40 % identity), our repertoire mining results and experimental validation with recombinant mAbs are consistent with previous work indicating that vaccination with 09 MIV produced a positive selection of *IGHV1-69*+ B cells [23, 24, 39], thus accounting for the differences in the cross reactivity induced by the two vaccines. Alternatively, *IGHV1-69* expressing B cells deletion as the result of 08/09 TIV immunization, as in transgenic mice expressing an anti-influenza virus BCR [4] cannot be ruled out.

The increase in the clonal frequency of *IGHV3-7* was observed in some individuals only in response to 09 MIV (Fig. 4a and c). This segment has been involved in the homotypic neutralization of 2009 pdmH1N1 and the 1918 A(H1N1) viruses, through recognition of the globular domain, evidencing the structural similarity between both pandemic viruses [48, 49]. Accordingly, an increase in the clonal frequency of *IGHV3-7* was observed in participants with homotypic neutralization, but not in individuals that showed heterotypic neutralization (Figs. 4, 5, and 8).

Also noteworthy is the finding that 09 MIV was associated with lower levels of somatic hypermutation, measured as the proportion of SHM in the V_H_ region per lineage. This effect contrasted with the increase of SHM proportion 7 days post vaccination with both 2008/09 and 2014/15 TIV. Low SHM rates are characteristic of rapid T-cell independent antiviral IgG responses in mice [40] and humans [41]. Accordingly, the expansion of *IGHV1-69* as a result of 09 MIV immunization together with low SHM rates may indicate an extra-follicular or germinal center-independent IgG B cell response that contributes to cross-reactivity in addition to a recall response towards common antigenic determinants shared between the pre- and the pandemic influenza strain.

Although the analysis of IGHV usage frequency revealed information that it is relevant to understand the response of the B cell repertoire to seasonal and pandemic vaccines, the absence of correlation between the frequency of *IGHV1-69* and heterotypic seroconversion is consistent with the finding that other IGHV segments may participate in heterotypic neutralization. Accordingly, we identified a *IGHV3-33* expansion in one individual that presented heterotypic seroconversion (Fig. 8b and d). The *IGHV3-33* segment shares a high sequence identity to *IGHV3-30* used in the pan-influenza FI6 broadly neutralizing antibody [45]. We have demonstrated that the observed clonal expansions are indeed, influenza virus specific (although not necessarily neutralizing) and in the case of the i04.1-69 *IGHV1-69* antibody, we demonstrated its reactivity to both 2008/09 TIV and 09 MIV.

Our results indicate that the application of vaccines with the 2009 pdmH1N1 virus whose HA had not circulated among the population for decades induced the production of antibodies against common antigenic determinants of the influenza virus that account, at least partially, for the heterosubtypic seroconversion and possibly for the cross-reactivity against various influenza virus subtypes. No evidence of OAS was found 7 days post immunization with the tested vaccines, although we cannot rule out that dominant clontoypes for the first vaccine that could be suboptimally expanded by MIV and TIV 14/15 were missed due to insufficient sequencing depth. Moreover, the low rates of somatic hypermutation as a result of 09 MIV immunization, as compared to TIV, could indicate an ‘innate’ extra-follicular response lead by non-antigen experienced B cells [54] and that contributes to the cross-reactivity induced by 09 MIV immunization or natural infection with 2009 pdmH1N1.

Research on the structural complexity of the repertoire of B cells in response to the structural complexity and variability of influenza viruses may contribute to evaluate experimental vaccines in terms of clonal competition, and for the identification of those epitopes that induce broadly neutralizing antibodies required for a universal vaccine capable of preventing infections by a large number of virus subtypes, including new variant viruses with pandemic potential.

## Conclusion

Immunization with different influenza viral strains produce distinctive effects in terms of cross-neutralization and the peripheral B cell repertoire, which are related with the degree of exposures to such variants. The factors that appear to contribute to this effects are the existence of conserved epitopes within different viral strains, however other factors such as a primary non-germinal center differentiation pathway against the new variants may also play a role.

## Additional files

Additional file 1:
**Light chain genes used for the production of recombinant monoclonal antibodies.** (XLSX 12 kb) Additional file 2:
**Serologic analysis of 08/09 TIV and 09 MIV 2009 response.** Hemagglutination inhibition assay (HAI) and microneutralization assay (MN) for A(H1N1) 2008, A(H3N2) 2008, and AH1N1pdm 2009 for 08/09 TIV and 09 MIV. Titer is shown in the *y* axis for pre-immune, 7, 14, and 30 days post vaccination in the *x* axis. (PDF 357 kb) Additional file 3:
**Sequencing and repertoire mining metrics with ImmunediveRsity [** 30 **] by vaccination trial and by individual.** (XLSX 23 kb) Additional file 4:
**Day 7 post vaccination/day 0 (** ***D*** ^***SW***^ **d7/** ***D*** ^***SW***^ **d0) Shannon-Weaver index ratio (** ***D*** ^***SW***^ **) for clonotypes (a) and lineages (b) in response to vaccination with TIV and 09 MIV indicates that the 09 MIV vaccine induced an increase in the diversity of the repertoire on day 7 after vaccination, while TIV induced a reduction in the diversity (Mann–Whitney test.** ***P* <0.01). (PDF 23 kb) Additional file 5:
**Pre- and post-vaccination SHM rates in selected** ***IGHV*** **segments according to vaccination trial.** Dots depict mutation rate in V_H_ region (%) per lineage. A total of 670 randomly sampled lineages per individual were selected, filtered according *IGHV* use, and plotted. (Kruskal-Wallis test. Dunn’s correction for multiple testing. ***P* <0.01, ****P* <0.001). (PDF 565 kb) Additional file 6:
**Per individual effect of immunization with 09 MIV on the repertoire of peripheral B-lymphocytes.** Changes in the relative frequency of use of IGHV segments (Δ day 7 – day 0) for individuals with homotypic seroconversion (blue), and with heterosubtypic seroconversion (red) for 47 IGHV segments. IGHV usage is expressed as the proportion of clonotypes using a particular IGHV segment. (PDF 230 kb) Additional file 7:
**CDRH2 sequence in large** ***IGHV1-69*** **-expressing V**_**H**_ **clonotypes.** Sequence logos [55] of CDRH2 (IMGT numbering) of the three largest *IGHV1-69* V_H_ clonotypes per individual classified according to (a) homotypic and (b) heterotypic seroconversion, as well as according to (c) no expansion (Δ day 7 – day 0 < 1 standard deviation (3.4)) or expansion (Δ day 7 – day 0 > 1 standard deviation (3.4)). (PDF 35 kb)

## Abbreviations

BCR: B cell receptor
CDRH2: Complementarity determining region heavy 2
CDRH3: Complementarity determining region heavy 3
d0: Day 0
d7: Day 7
*D*^*SW*^: Shannon-Weaver index
HA: Hemagglutinin
HIA: Hemagglutination inhibition assay
MIV: Monovalent influenza vaccine
MN: Microneutralization test
OAS: Original antigenic sin
PCA: Principal component analysis
pdmH1N1: Pandemic influenza H1N1
SHM: Somatic hypermutation
TIV: Trivalent inactivated vaccine
V_H_: Heavy variable region
V_L_: Light variable region
