# Supplementary figures and images for: Longitudinal analysis of the peripheral B cell repertoire reveals unique effects of immunization with a new influenza virus strain
Source: Genome Med. 2015 Nov 25;7:124. doi: 10.1186/s13073-015-0239-y (PMC4658769; doi:10.1186/s13073-015-0239-y)

2009 pdmH1N1

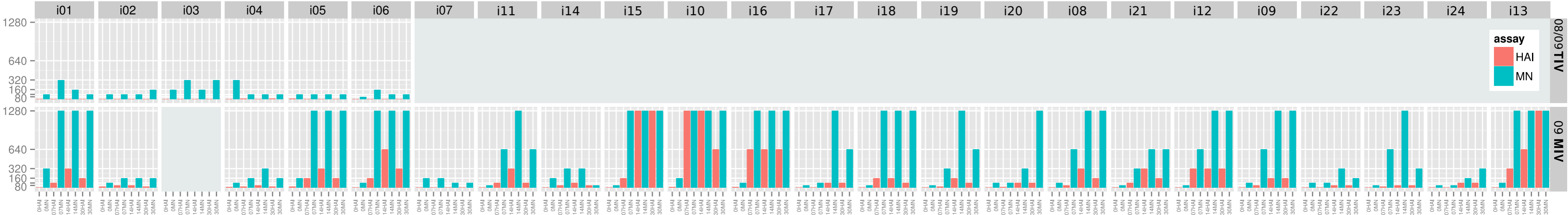

2008/2009 H1N1

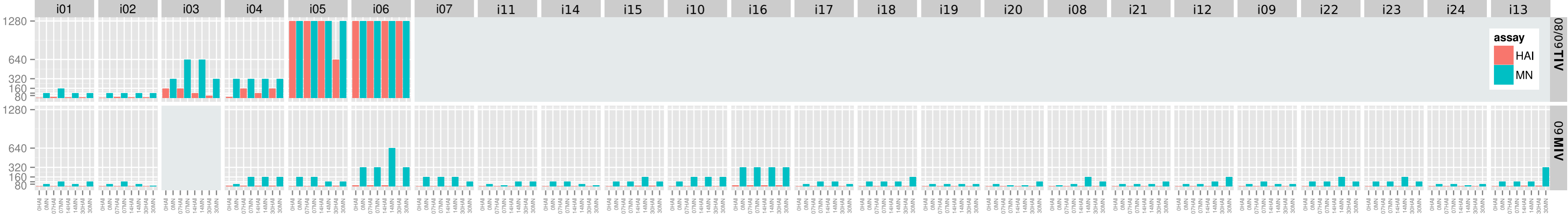

2008/2009 H3N2

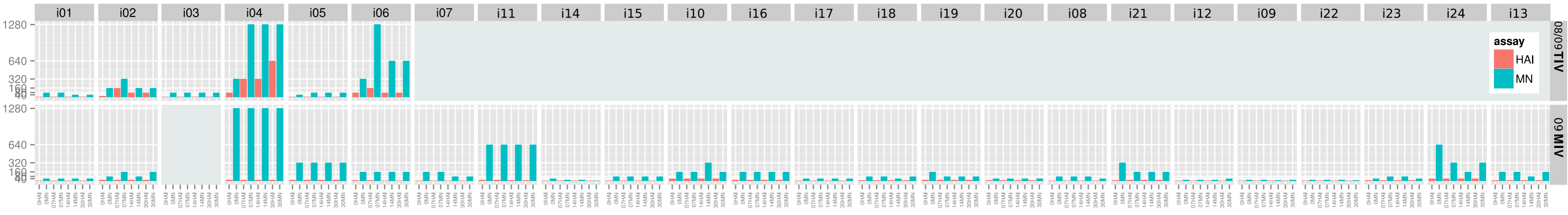

Days

Supplement: Additional file 2: — Serologic analysis of 08/09 TIV and 09 MIV 2009 response. Hemagglutination inhibition assay (HAI) and microneutralization assay (MN) for A(H1N1) 2008, A(H3N2) 2008, and AH1N1pdm 2009 for 08/09 TIV and 09 MIV. Titer is shown in the y axis for pre-immune, 7, 14, and 30 days post vaccination in the x axis. (PDF 357 kb) [file 13073_2015_239_MOESM2_ESM.pdf]

A

 $V_H$  Clonotypes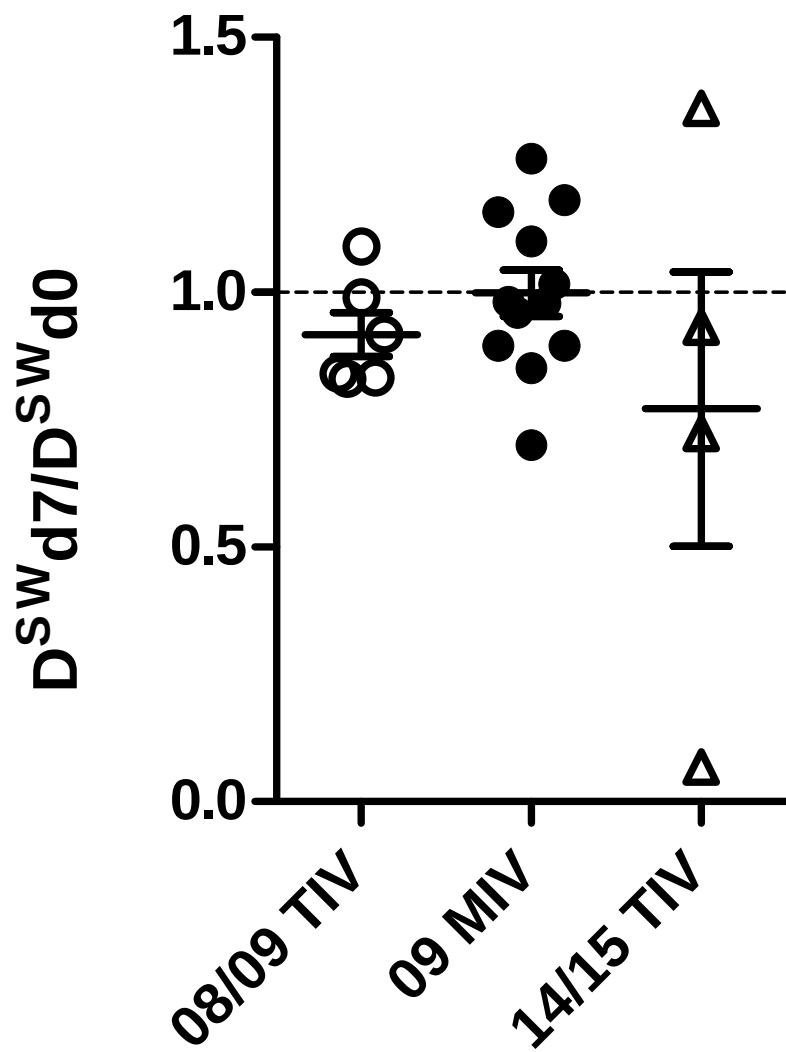

B

 $V_H$  Lineages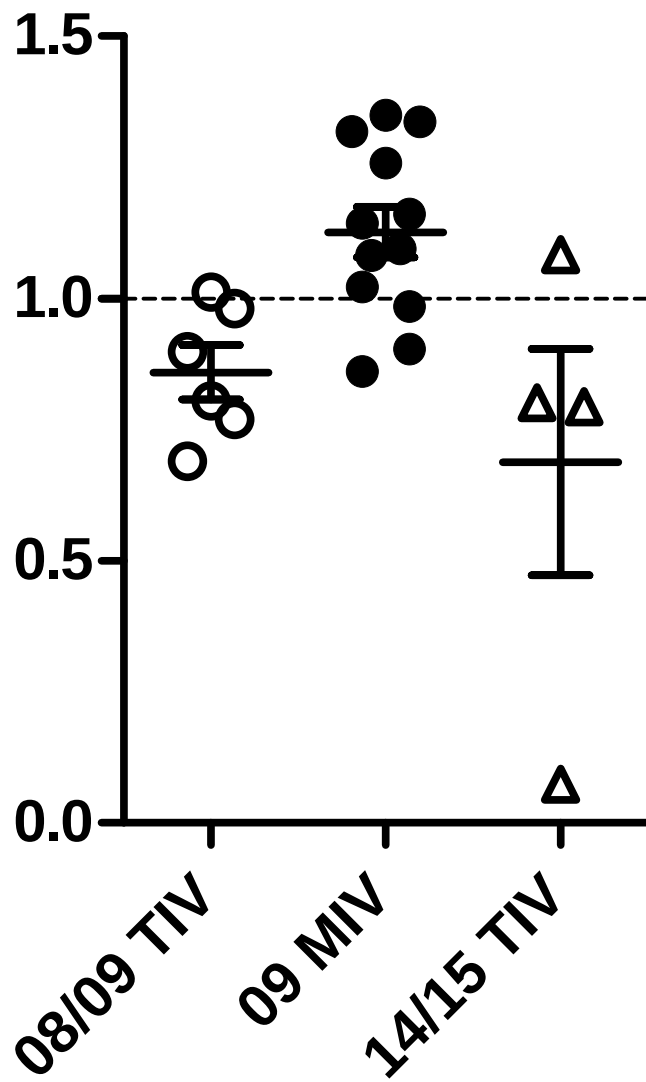

Supplement: Additional file 4: — Day 7 post vaccination/day 0 ( D SW d7/ D SW d0) Shannon-Weaver index ratio ( D SW ) for clonotypes (a) and lineages (b) in response to vaccination with TIV and 09 MIV indicates that the 09 MIV vaccine induced an increase in the diversity of the repertoire on day 7 after vaccination, while TIV induced a reduction in the diversity (Mann–Whitney test. **P <0.01). (PDF 23 kb) [file 13073_2015_239_MOESM4_ESM.pdf]

**A**

IGHV1-69

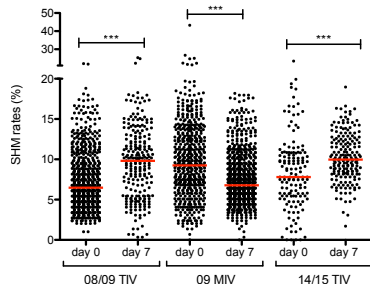**B**

IGHV3-7

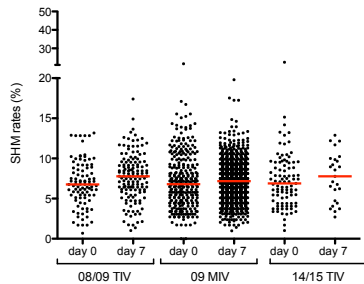**C**

IGHV4-39

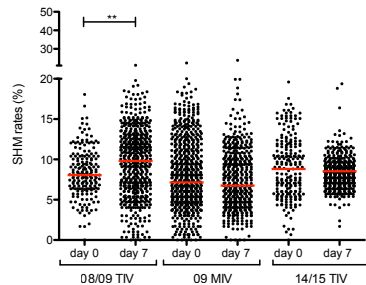**D**

IGHV1-3

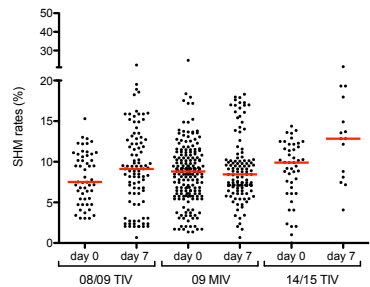**E**

IGHV3-15

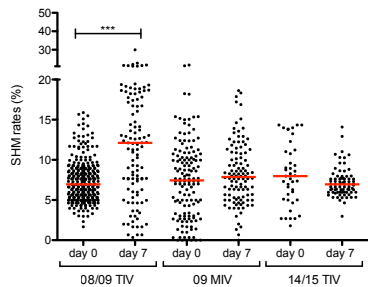**F**

IGHV4-59

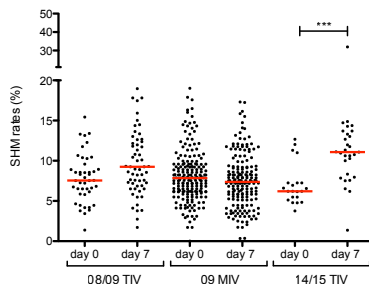

Supplement: Additional file 5: — Pre- and post-vaccination SHM rates in selected IGHV segments according to vaccination trial. Dots depict mutation rate in VH region (%) per lineage. A total of 670 randomly sampled lineages per individual were selected, filtered according IGHV use, and plotted. (Kruskal-Wallis test. Dunn’s correction for multiple testing. **P <0.01, ***P <0.001). (PDF 565 kb) [file 13073_2015_239_MOESM5_ESM.pdf]

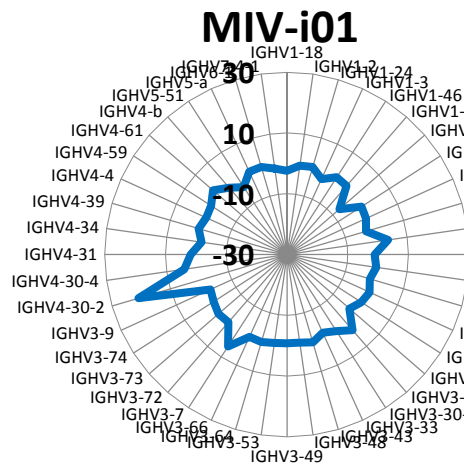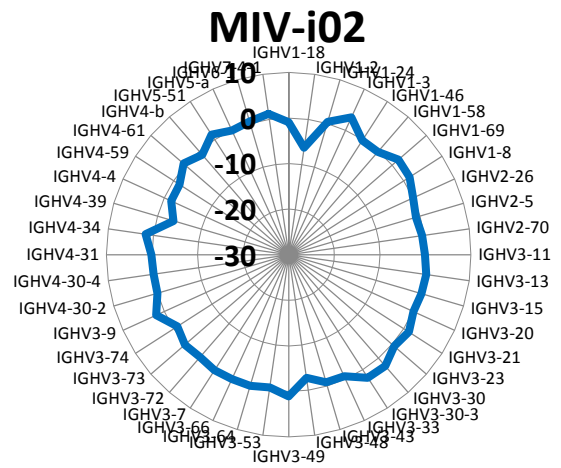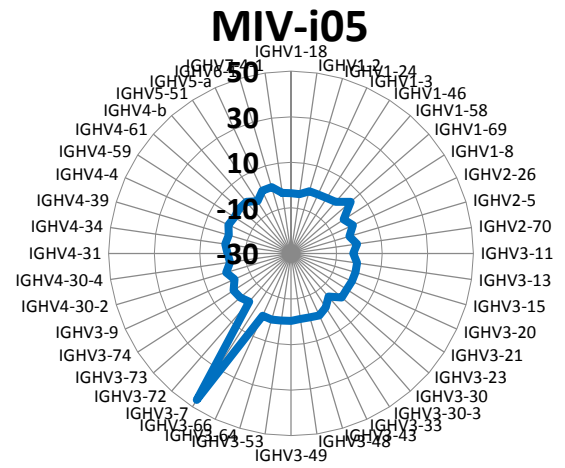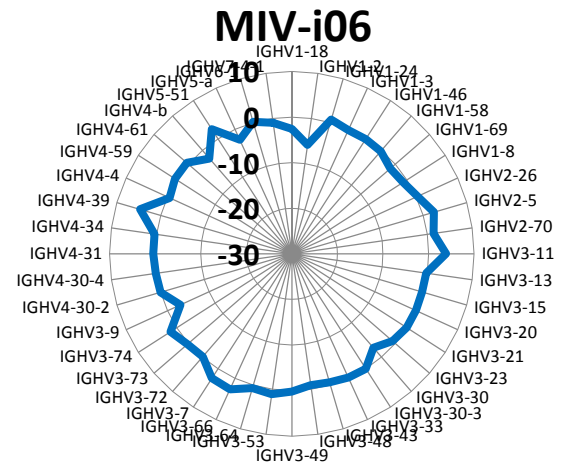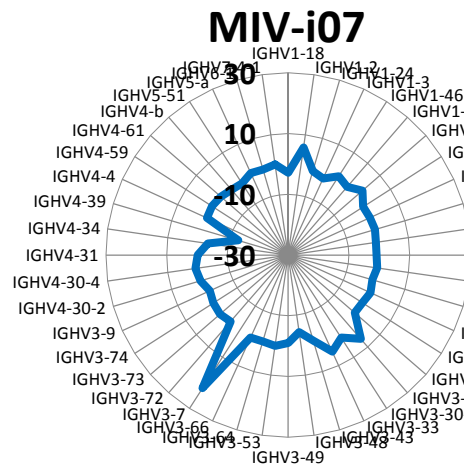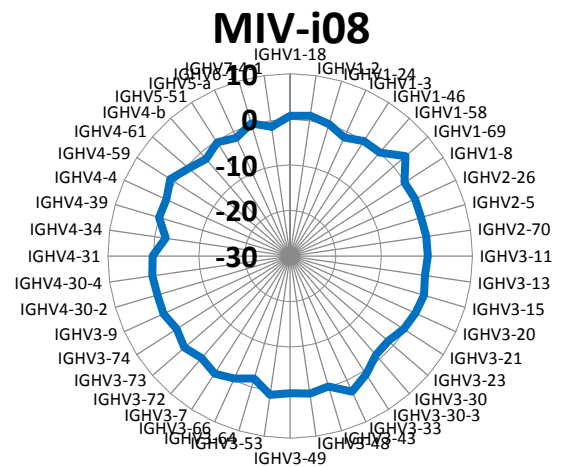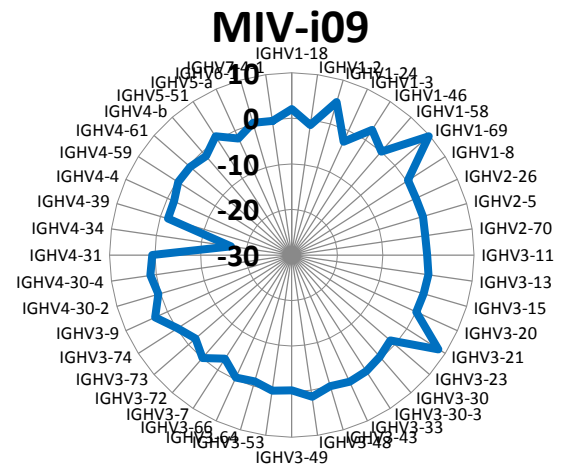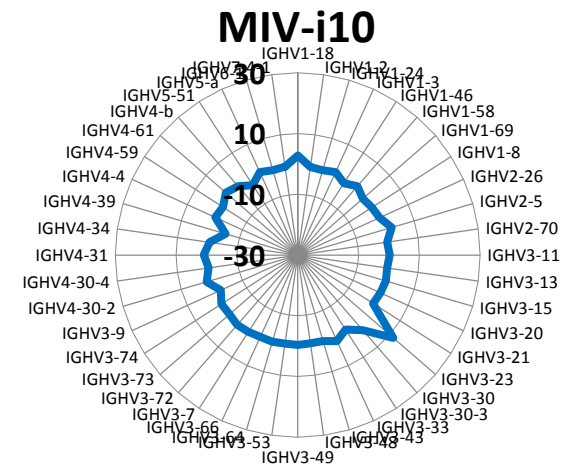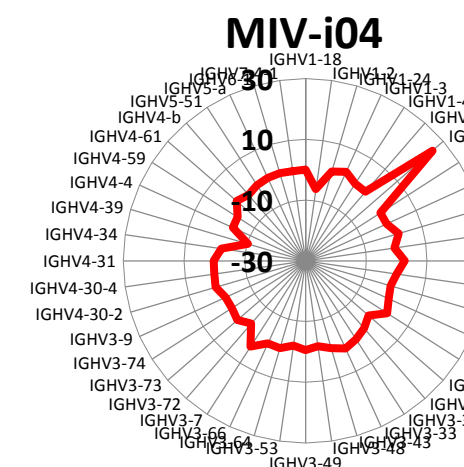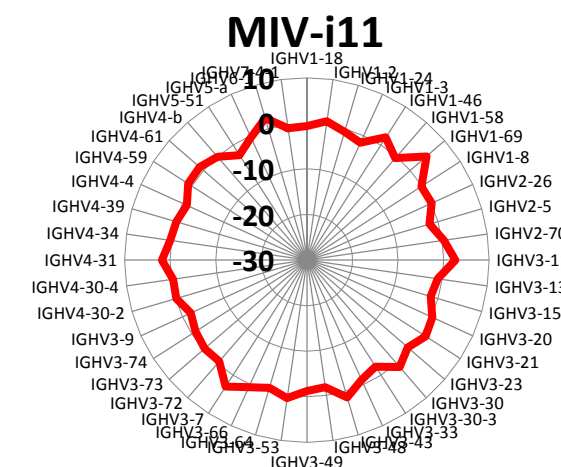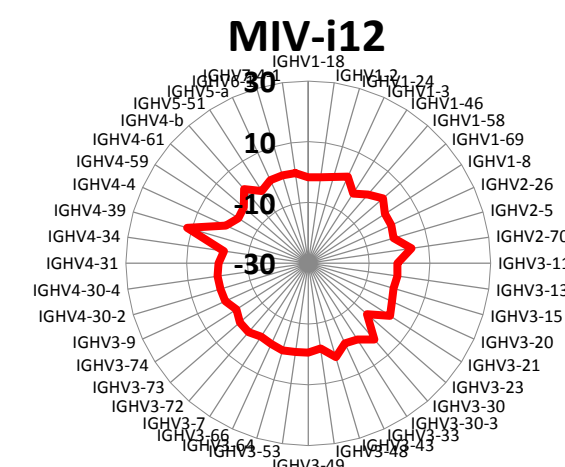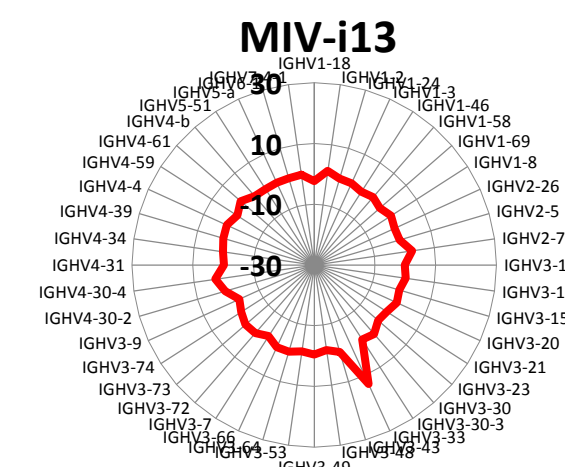

Supplement: Additional file 6: — Per individual effect of immunization with 09 MIV on the repertoire of peripheral B-lymphocytes. Changes in the relative frequency of use of IGHV segments (Δ day 7 – day 0) for individuals with homotypic seroconversion (blue), and with heterosubtypic seroconversion (red) for 47 IGHV segments. IGHV usage is expressed as the proportion of clonotypes using a particular IGHV segment. (PDF 230 kb) [file 13073_2015_239_MOESM6_ESM.pdf]
