# Supplementary material for: Longitudinal analysis of the peripheral B cell repertoire reveals unique effects of immunization with a new influenza virus strain
Source: Genome Med. 2015 Nov 25;7:124. doi: 10.1186/s13073-015-0239-y (PMC4658769; doi:10.1186/s13073-015-0239-y)
Supplement: Additional file 7: — CDRH2 sequence in large IGHV1-69 -expressing V H clonotypes. Sequence logos [55] of CDRH2 (IMGT numbering) of the three largest IGHV1-69 VH clonotypes per individual classified according to (a) homotypic and (b) heterotypic seroconversion, as well as according to (c) no expansion (Δ day 7 – day 0 < 1 standard deviation (3.4)) or expansion (Δ day 7 – day 0 > 1 standard deviation (3.4)). (PDF 35 kb) [file 13073_2015_239_MOESM7_ESM.pdf]

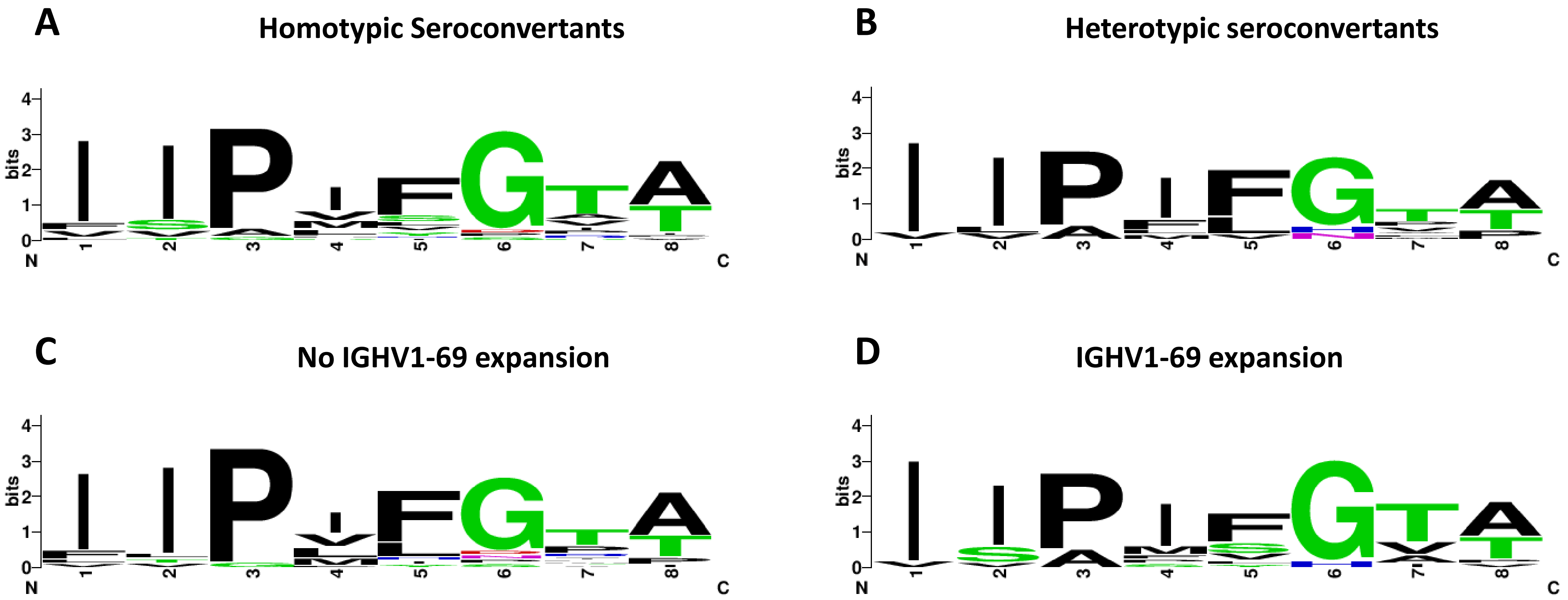
A
Homotypic Seroconvertants
B
Heterotypic seroconvertants
C
No IGHV1-69 expansion
D
IGHV1-69 expansion
bits
0
1
2
3
4
N
C
1
2
3
4
5
6
7
8
